# Mixing Time, Inversion and Multiple Emulsion Formation in a Limonene and Water Pickering Emulsion

**DOI:** 10.3389/fchem.2018.00132

**Published:** 2018-05-04

**Authors:** Laura Sawiak, Katherine Bailes, David Harbottle, Paul S. Clegg

**Affiliations:** ^1^School of Physics and Astronomy, University of Edinburgh, Edinburgh, United Kingdom; ^2^School of Chemical and Process Engineering, University of Leeds, Leeds, United Kingdom

**Keywords:** droplet, interface, emulsification, colloid, cluster

## Abstract

It has previously been demonstrated that particle-stabilized emulsions comprised of limonene, water and fumed silica particles exhibit complex emulsification behavior as a function of composition and the duration of the emulsification step. Most notably the system can invert from being oil-continuous to being water-continuous under prolonged mixing. Here we investigate this phenomenon experimentally for the regime where water is the majority liquid. We prepare samples using a range of different emulsification times and we examine the final properties in bulk and via confocal microscopy. We use the images to quantitatively track the sizes of droplets and clusters of particles. We find that a dense emulsion of water droplets forms initially which is transformed, in time, into a water-in-oil-in-water multiple emulsion with concomitant changes in droplet and cluster sizes. In parallel we carry out rheological studies of water-in-limonene emulsions using different concentrations of fumed silica particles. We unite our observations to propose a mechanism for inversion based on the changes in flow properties and the availability of particles during emulsification.

## Introduction

That emulsions can turn “inside out” with prolonged mixing is a phenomenon which has been exploited in butter making for millennia. In the butter churn the fat droplets from cream become the continuous medium (Rønholt et al., 2013). The process is quite complex, involving very many components some of which are lost to the buttermilk. The same phenomenon has been noted much more recently, under high shear, for particle-stabilized emulsions. These emulsions are simpler: they incorporate only three components, most commonly none of them is close to its solidification temperature. In spite of its simplicity this type of inversion process is not yet fully understood. Without this understanding predictive control of emulsification using particles is not possible.

Particle-stabilized emulsions (also called Pickering emulsions) have droplet interfaces stabilized by colloidal particles rather than molecular surfactants (Binks and Horozov, 2006; Ngai and Bon, 2015). Preparing a stable Pickering emulsion involves optimizing the particle wettability, the liquid and particle concentrations, the order of ingredient addition, the liquid flow properties and the mixing protocol. At different points within this highly multi-dimensional phase space a whole slew of different types of emulsions can be fabricated (Vignati et al., 2003; Destribats et al., 2012; Clegg et al., 2016; Domenech and Velankar, 2017) and, by varying one or more of the control variables, transitions between emulsion types are observed (Binks et al., 2010; French et al., 2016). The simplest Pickering emulsions are made up of dispersed droplets covered by a more-or-less complete layer of particles. Droplet coalescence is prevented by the mechanical barrier provided by the particles. Early on in Pickering emulsion research it was observed that inversions between water-in-oil and oil-in-water Pickering emulsions could be achieved by either changing the wettability of the particles (transitional inversion) or, in some cases, by changing the proportion of the two liquid phases (catastrophic inversion) (Whitby and Wanless, 2016). Subsequently, the number of inversion routes discovered has multiplied.

Crucial aspects of Pickering emulsion inversion behavior were revealed in a study of perfume oils emulsified using fumed silica particles (Binks et al., 2010). This research built on the existing understanding of transitional and catastrophic inversion; in particular, the authors were aware that dispersing the particles in one solvent would bias the system to form an emulsion with that solvent as the continuous phase. In order to minimize this effect, they sprinkled the particles between the two phases prior to emulsification. Alongside confirming transitional and catastrophic emulsion behavior for industrially relevant oils, this study also highlighted the fact that some of these Pickering emulsions could be induced to invert by either increasing the concentration of particles or by increasing the emulsification time. It was suggested that the clusters of particles typical of a high particle concentration/short mixing time sample were more hydrophobic than the well-dispersed particles seen at lower concentrations/longer mixing times. This effect was presented in most detail for a system of water, limonene and fumed silica particles in which the liquid volumes were equal. For example, in one study water droplets in oil form at short times, these grow in size and become oil-in-water-in-oil droplets before finally undergoing an inversion to oil-droplets in water after several minutes of high shear. Below we study related behavior in a somewhat different composition range where we have the added benefit that we are able to observe the size of silica clusters and the details of the droplet size distribution.

Choosing the appropriate combination of mixing time, shear rate and particle concentration is also an important part of designing a stable emulsion in the limit of very high concentrations of droplets. Such systems are called high internal phase emulsions (HIPEs); in Zang and Clegg (2013) the dense emulsions were made up of water droplets stabilized by fumed silica particles. This system can be destabilized into an oil-in-water Pickering emulsion by using too few particles, shearing too fast or for too long. Using confocal microscopy and analysis of the bulk emulsion composition it was possible to show that, in the formation of a HIPE, both droplet size and excess water decrease with time. Provided that the particle concentration is sufficiently high, water droplets are gradually packed into the oil continuous phase even though it is already well populated. It was tentatively suggested that this occurs if the oil phase is sufficiently viscous. Hence oil droplet formation occurs at low particle concentration, after long mixing times or at high shear rates because, under these conditions, the viscosity of the oil phase has markedly decreased.

Close to the point at which an emulsion undergoes inversion it is quite common to observe the formation of a multiple emulsion, i.e., droplets of one liquid phase inside droplets of another. The region of the multi-dimensional parameter space where this occurs is of fundamental interest because it represents the point at which the opposite mean curvatures of the same interfaces are captured in the same sample. It is also of interest for applications because it holds the promise of a simple route to preparing these relatively complex samples. For example, a water-in-oil-in-water emulsion can be used to reduce the fat content of foods or to address a variety of encapsulation challenges. The absence of a simple mixing protocol for preparing multiple emulsions with long-term stability is currently a barrier to their exploitation (Clegg et al., 2016). Very recently a generic pathway for the creation of oil-in-water-in-oil multiple emulsions has been proposed based on phase inversion (Kim et al., 2018). Here we consider one scenario for the production of water-continuous multiple emulsions.

Below we present experimental results on the emulsification of combinations of water, limonene and fumed silica particles. We target the behavior when the aqueous phase is in the majority (φ_w_ = 0.8). Qualitatively, we show using bulk observations combined with fluorescence confocal microscopy that a dense emulsion of water droplets rapidly forms which undergoes an inversion after tens of minutes of high shear. The inverted emulsion is a water-in-limonene-in-water multiple emulsion. Quantitatively, we use image analysis to show the complex evolution of the water-droplet size and the size distribution of particle clusters as inversion is approached. We find that the droplets bifurcate into a dense population of small droplets and a small number of very large water droplets which go on to become the continuous phase after inversion. We separately study the flow properties of dense populations of small droplets to support our proposed explanation of the inversion process.

## Materials and methods

### Materials

The fumed silica, type H3O, is partially hydrophobic and was a gift from Wacker, the manufacturing company. Fumed silica is formed of primary particles 30 nm in diameter which aggregate irreversibly into group sizes of 100–1,000 nm. These often cluster further to give final sizes in the micron range. The oil, limonene (R-+, 97%, Sigma-Aldrich), was filtered with activated alumina three times to remove polar impurities. Water was distilled and then passed through a Milli-Q machine giving an initial resistivity of 18 MΩ cm.

### Preparation of emulsions for phase inversion studies

The powdered particle method from Binks et al. (2010) was used to minimize the extent to which the system is biased to being either oil or water-continuous. The densest phase, water, was put in the vial first. Then a known amount of silica was sprinkled on top before limonene was finally added. Emulsions had a water volume fraction of 80%, and a silica weight fraction of 0.5%. Nile red was added to the water phase before it was measured out. The concentration of dye in the water was 2.1 μM. A Polytron PT 3100 rotor stator with a 12 mm head was used at 10,300 rpm to emulsify each sample for varying lengths of time. Emulsification durations of 1, 5, 10, 15, 20, and 25 min were used for a series of separate samples.

Preparation of water-in-oil emulsions for rheology studies: To make water-in-oil (w/o) emulsions, which are free of particle clusters and have no tendency to undergo inversion, a dispersion of silica in limonene was first prepared. A known amount of fumed silica was added to a given volume of limonene with dispersion being performed via three cycles of first 2 min. vortex mixing and second ultra-sonication using a probe (VCX 500, Sonics and Materials Inc., Newtown USA) for 2 min. at 20% maximum amplitude. The 25 mL emulsions were made up with a water volume fraction of 0.7. The silica weight fractions were 0.11, 0.19, 0.27, 0.35, 0.44, and 0.52% w/w respectively. A Silverson L5M-A mixer was used to homogenize each sample for 10 min at 10,000 rpm.

### Rheology experiments

A rheology study was carried out using a TA Instruments DHR-2 rheometer using a Couette geometry. Here, the bob length = 42 mm, bob diameter = 28 mm, cup diameter = 30.4 mm. The freshly loaded sample was allowed to rest for 120 s. This was followed by a frequency sweep for which the stress was 1.0 Pa and the angular frequency varied from 0.3 to 100 rad/s. For each step, there were 5 conditioning cycles, and 5 acquisition cycles. This was followed by an upsweep in shear stress from 0.9 to 200 Pa in order to measure a flow curve with an equilibration time of 5 s, and an acquisition time of 20 s.

### Optical microscopy

To characterize w/o emulsions, an optical microscope was used with a ×20 objective, Olympus BX51 with a GXCAM HiChrome-MET (GT Vision) camera system. A curved spatula was used to scoop some emulsion onto a cover slip for observation. Droplet sizing was done using Matlab. More than 100 droplets from each sample were measured by diameter.

### Confocal fluorescence microscopy

A Zeiss Observer.Z1 inverted microscope in conjunction with a Zeiss LSM 700 scanning system was used to visualize emulsions following various emulsification times. For confocal microscopy studies, the limonene was dyed with Nile Red (2.1 μM, Technical Grade, Sigma-Aldrich). Deployment of two channels allowed separate visualization of dyed oil, and silica in the presence of dyed oil. The first channel was for fluorescence from molecules excited using a laser with wavelength 488 nm and second channel for 555 nm. A short pass filter at 555 nm and a dichroic mirror was used to separate the light for these two channels. A spatula was used to scoop some of each emulsion onto a cover slip for examination. For phase inverted samples, a pipette was used instead.

## Results and discussion

### Macroscale

**Figure 1:**
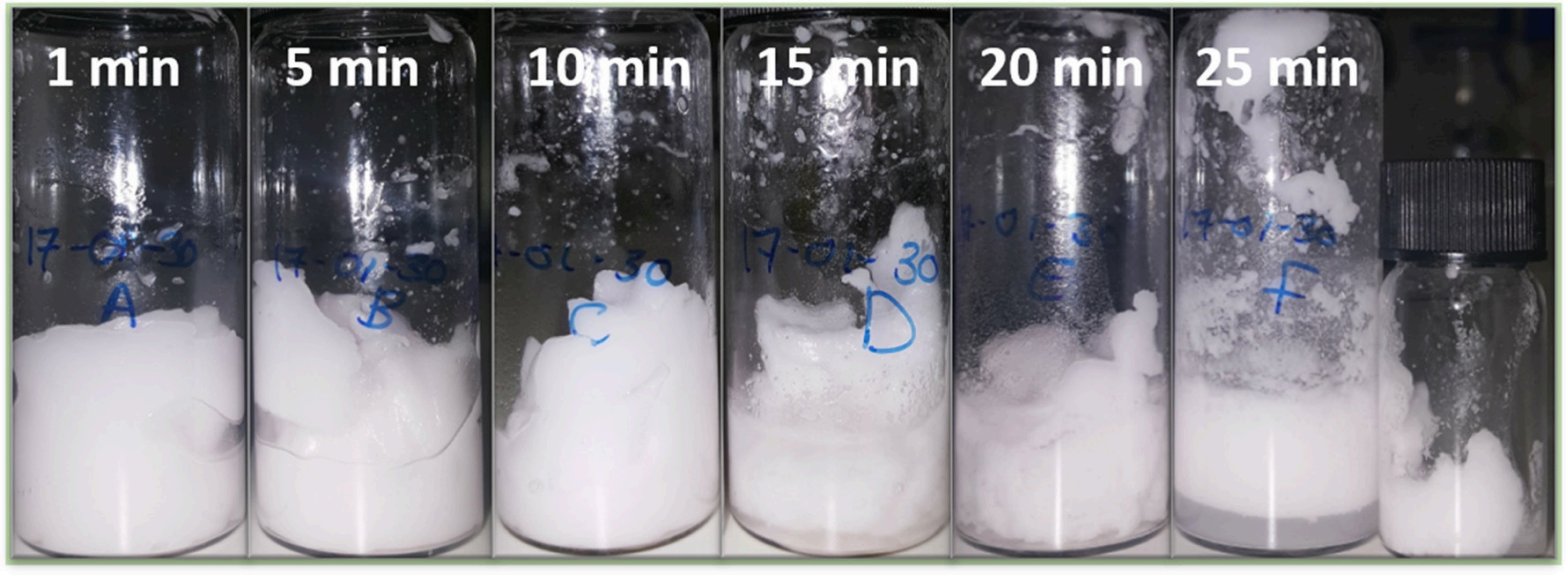
Six different emulsion samples created by high shear mixing for different periods of time. The samples are 80 vol.% water and the oil is limonene. Initially a dense emulsion of water droplets is formed stabilized by fumed silica particles. After 15 min a continuous phase of water is beginning to become visible at the base of the sample. The final smaller vial contains dense gelled droplets recovered from the rotor stator head after 25 min.

Figure 1 shows six separate emulsions prepared by high-shear mixing for different periods of time. These vials all contain water, limonene and silica particles with water and silica fractions 80 vol% and 0.5 wt% respectively. Each vial was emulsified for a different period of time indicated by the numbers in each frame. After 1 min of high shear mixing the sample is fully emulsified and appears white and gel-like. As will be confirmed using fluorescence confocal microscopy below, the sample is full of water droplets indicating that it is a particle-stabilized high internal phase emulsion (HIPE). Once the samples had begun to gel it was necessary to move the vial around to give a stirring effect in addition to the localized high shear.

The images of the vials at 15 and 20 min evidence the presence of excess water at the base of the vials. Hence, it is likely that inversion has begun to occur in some parts of the sample. Subsequently, the emulsion begins to yield more substantially and flows fast, before jammed droplets intermittently stop the flow. After a number of flow-stops, the emulsion flows easily throughout the sample. The result can be seen at 25 min of emulsification: the emulsion can now cream. The water is now the continuous phase and the emulsion is no longer a HIPE. The final small vial shows emulsion scraped off the rotor stator. This part of the emulsion had not inverted, and so still exhibits a yield stress.

### Microscale

**Figure 2:**
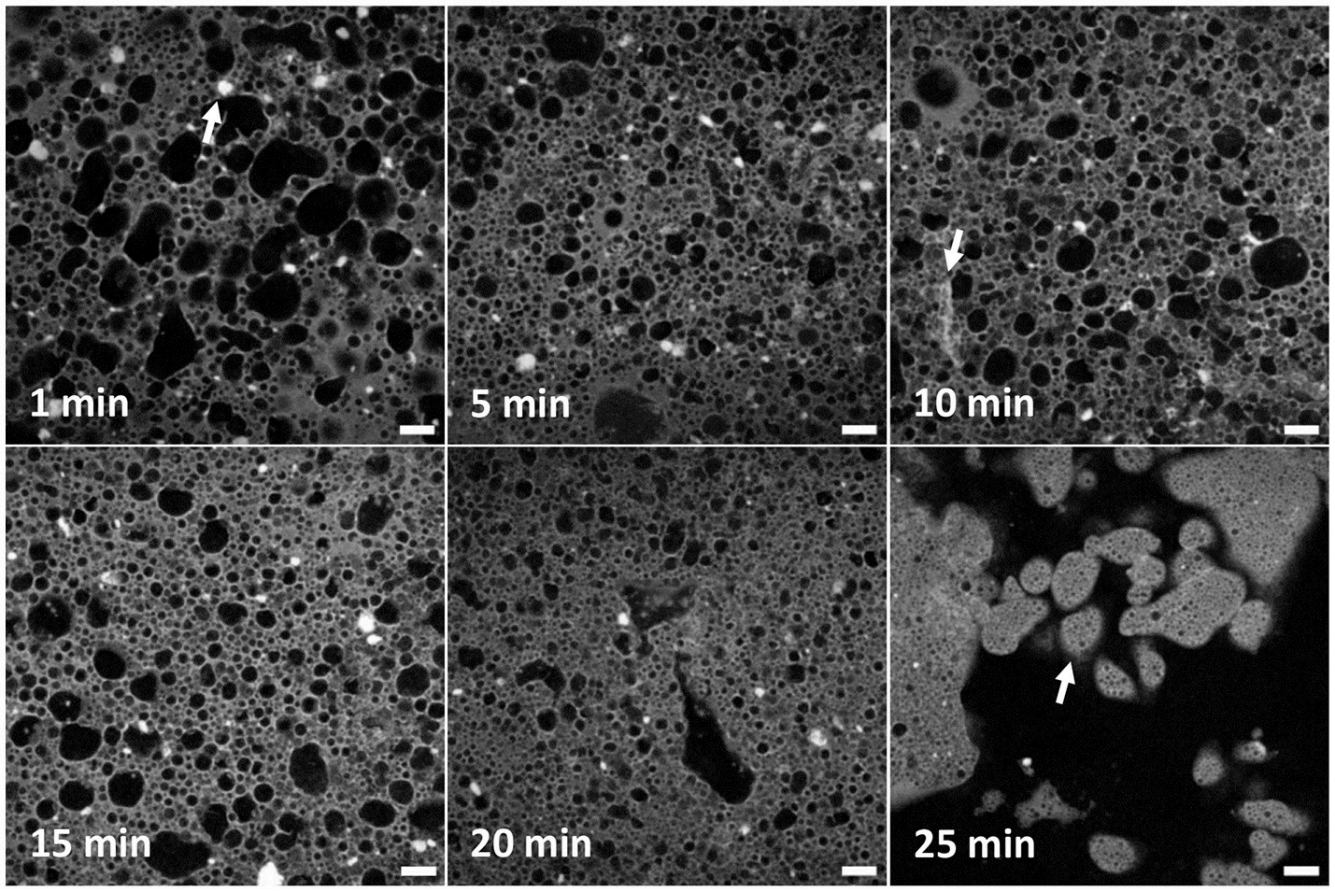
A series of confocal micrographs taken using a ×10 objective lens of different emulsion samples prepared by high-shear mixing for different periods of time. All scale bars are 100 μm. The darker phase is water and the lighter phase is limonene dyed with Nile Red. Clusters of poorly dispersed fumed silica are evident as solid bright regions. An example is given by an arrow in the first panel. The changes to the droplet and cluster size distribution are analyzed and discussed in the text. The second arrow shows an elongated structure of silica. The arrow in the final panel points to multiple emulsion droplets of water-in-oil-in-water.

Figure 2 shows typical fluorescence confocal micrographs, each corresponding to a particular mixing time, shown top left of each panel. Water (black), Nile Red dyed limonene (pale gray) and the silica (white) are all clearly visible via the merging of separate channels. These micrographs demonstrate that both the size and shape of droplets and clusters of particles change with emulsification time. In the panel at 1 min many non-spherical droplets can be seen reflecting both the dense packing of the droplets and the likely role of coalescence. Smaller droplets are also visible. By 5 min there is less difference in size between small and large droplets in general while the larger ones are more regular in shape than before. At 10 min the emulsion looks less uniform: there are some small droplets, some large droplets with a couple of droplets apparently bigger than at the 5 min point. At 15 min, the difference between big and small droplets continues to grow with the big droplets being few in number. In the 20 min panel only a few large droplets can be seen, the rest are small. Recalling Figure 1, at this point water has been expelled from the emulsion which presumably is the natural limit of large water droplets. At 25 min, the phase inversion is captured. No large droplets can be seen anymore, but the continuous phase can clearly be identified as water. Many multiple emulsion droplets can be seen as indicated by the white arrow in the final panel. The inner water droplets are very small, and uniform over large areas.

In addition to droplets, many clusters of silica can be seen (Figure 2, 1 min panel, as indicated by a white arrow). These have been speculated about previously (Binks et al., 2010) as outlined in the Introduction but are imaged here for the first time. The prevalence of these clusters reflects the manner in which the samples were created, i.e., pre-dispersal of particles was avoided. In the confocal micrographs the clusters are very bright and have a low aspect ratio. They appear both in the bulk oil phase and stuck to the interface of droplets. At 5 min, clusters appear smaller on average. This trend continues throughout the time sequence until only a handful of clusters can be seen in the final image. In the 10 min panel, near the bottom left corner an elongated structure can be seen as indicated by an arrow. Its brightness indicates that it is a structure of silica. It is less bright than other silica features and has a different shape to those before. It seems likely that these occur as a side-product of the other processes which lead to emulsion inversion. This will be discussed in more detail later.

**Figure 3:**
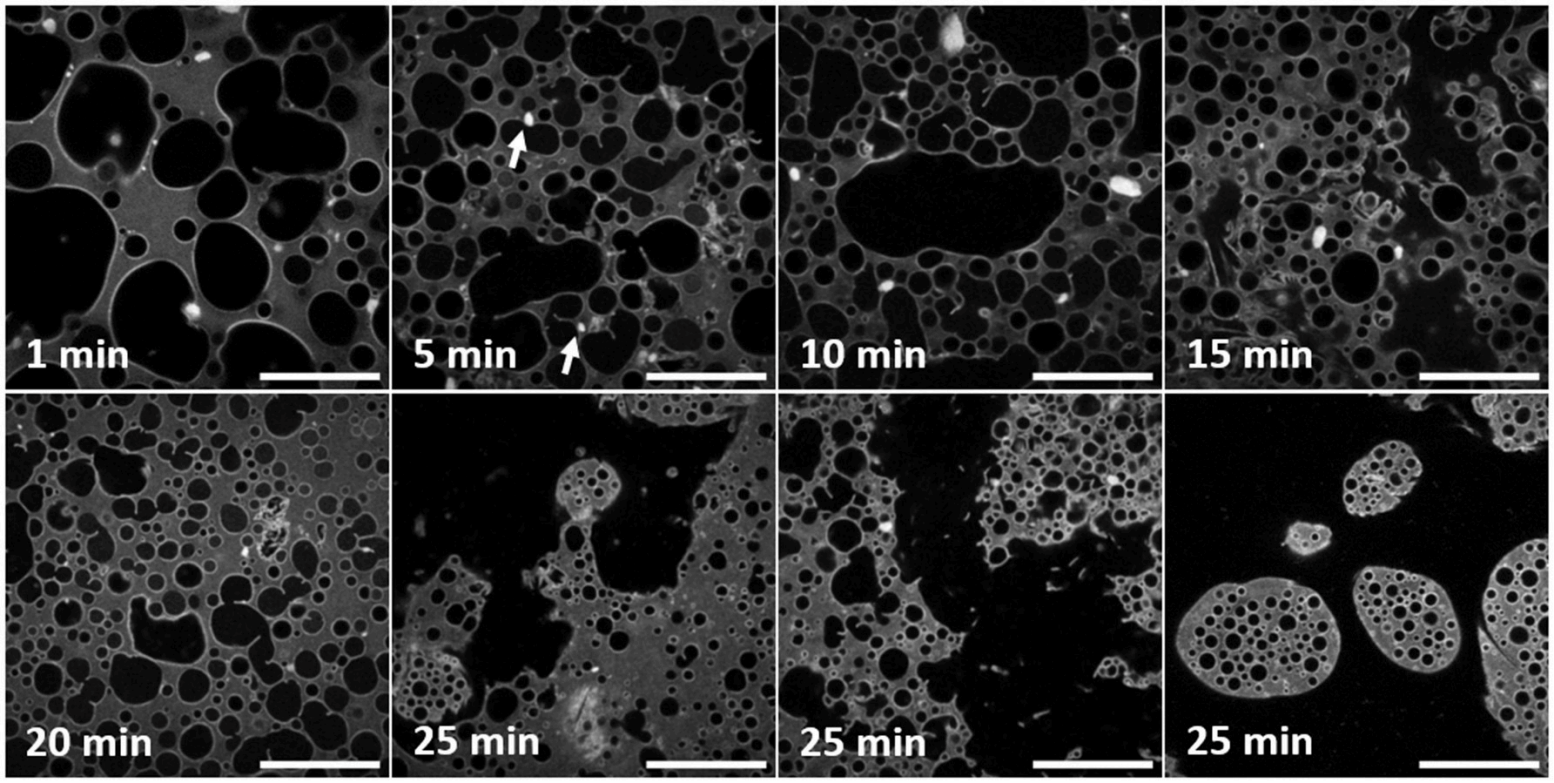
A series of confocal micrographs taken using a ×40 objective lens of different emulsion samples prepared by high-shear mixing for different periods of time. All scale bars are 100 μm. The darker phase is water and the lighter phase is limonene dyed with Nile Red. Both particles on droplet interfaces and clusters of particles are visible at this magnification. The changes to the droplet and cluster size distribution are analyzed and discussed in the text. The white arrows in the second panel point out examples of clusters of particles stuck to interfaces of droplets.

Figure 3 (objective ×40) reveals more details of the water droplets and particle clusters but over a smaller area, compared to Figure 2, which will be important for the quantitative analysis that follows. For the first 20 min these images confirm the droplet development described in relation to Figure 2. The three confocal micrographs corresponding to 25 min of high-shear mixing, Figure 3, demonstrate how heterogeneous the sample has become. The large and very irregular water spaces dominate the images. Within the water, there is floating debris. This is presumed to stem from the emulsification smashing up jammed regions of droplets. This section of water can be considered continuous. Pinch off events, where an interface bulges out in a spherical shape, before the neck of the bulge thins and breaks forming a new droplet are also suggested by these images. In the final 25 min confocal micrograph, multiple emulsion droplets can be seen. There are no large emulsion droplets. Phase inversion has occurred. The inner droplets are small and have a narrow size distribution.

Observing all of the particle clusters in Figure 3, the same trend as in Figure 2 can be seen. The higher resolution of these images highlights features invisible at lower magnification. Here, fewer bright regions of silica can be spotted and the density of particles on the droplet interfaces is reflected in brighter patches. At 5 min clusters are visible on the right-hand side of the image and stuck to droplets of varying size. Two examples are highlighted by white arrows. In the 15 min panel, brighter, straighter segments of silica can be seen. They appear near the boundary of large, irregularly shaped droplets. At 25 min, the edge of the multiple emulsion droplet visible at the top of the image has a textured interface.

**Figure 4:**
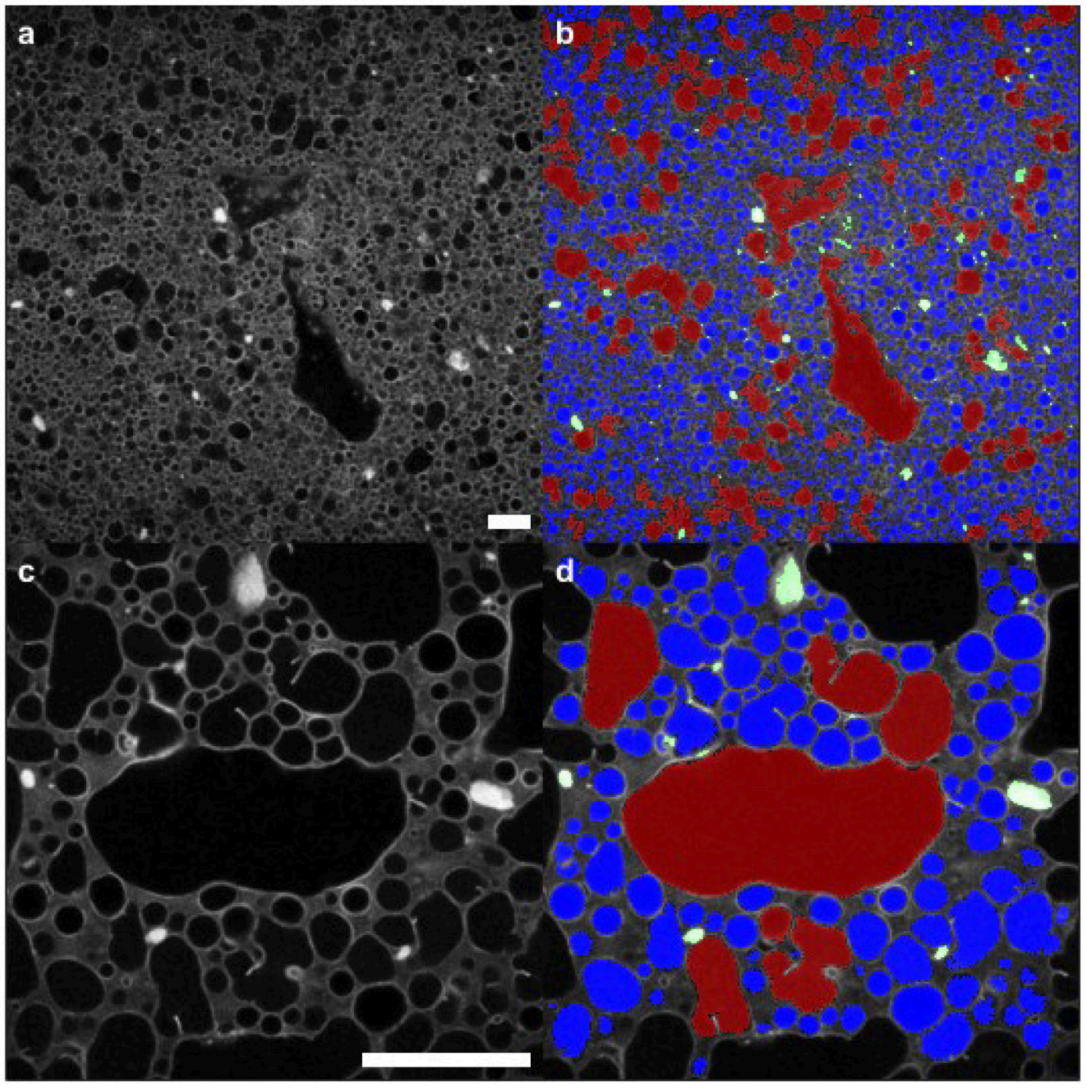
Segmentation of droplets and clusters of particles from the confocal image data. **(a,b)** images using ×10 objective (**c,d**) images using ×40 objective. Red signifies droplets larger than 20 μm in their largest dimension; blue indicates the smaller droplets. The segmentation procedure is described in the text; a selection of the resulting parameters are presented in subsequent images. Scale bars in **(a,c)** are 100 μm.

Figure 4 illustrates quantitative segmentation of the micrographs. On the left-hand side the originals are shown, after a Gaussian filter has been used to reduce random noise. At this point a threshold was selected by hand in order to separate the maximum number of droplets. Even with an optimum threshold, some droplets appear stuck together. A morphological opening was used to remove spurious bridges with a disk as the structuring element. The results can be seen in the right-hand side of this figure where the segmentation is overlaid in color. Red signifies droplets with a radius larger than 20 μm, while blue shows smaller droplets. Green indicates particle clusters. Throughout, objects touching the image border of the high resolution images were not included in the analysis. A similar procedure was used to segment the particle clusters which appear bright white in the micrographs.

**Figure 5:**
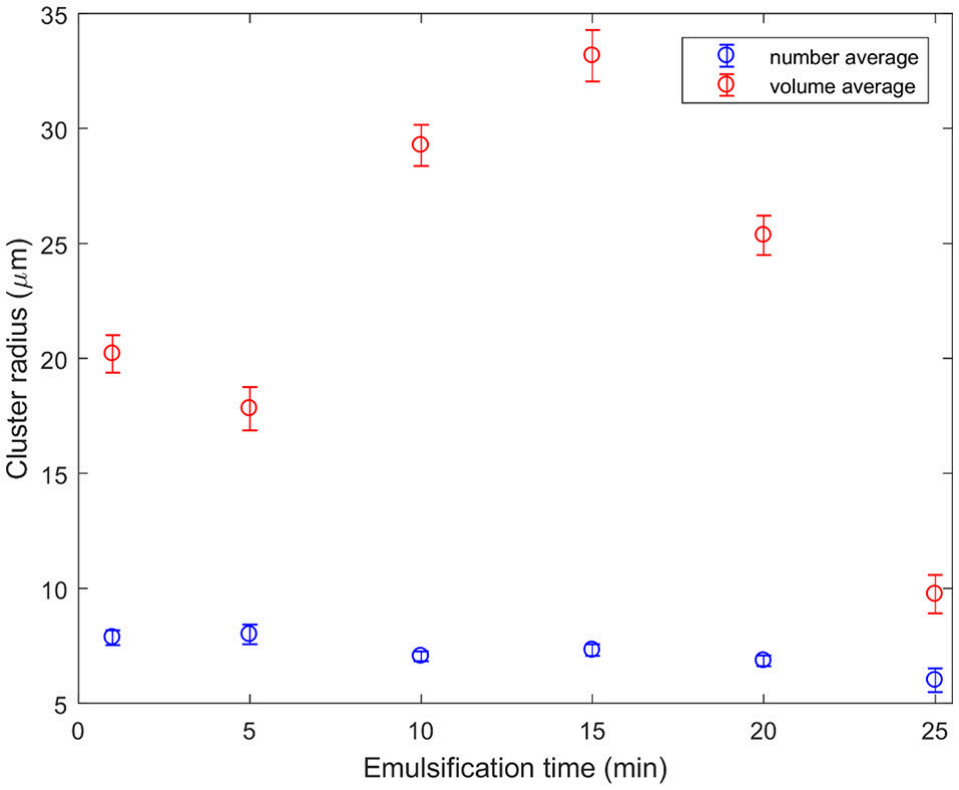
Particle cluster sizes segmented from the ×10 objective confocal micrographs as a function of emulsification time. The number average indicates that typical clusters are getting slightly smaller as they are sheared for longer. By contrast, a few very large clusters which appear to be created as large droplets fold over on themselves dominate the volume averages at intermediate emulsification times.

Figure 5 shows that normalization of cluster sizes matters. Normalization was carried out separately by volume or by number. Number averaging is an unweighted calculation of the mean. To find the volume-averaged diameter of a particle, d, the sum of d^4^ divided by the sum of d^3^ must be calculated. This method of averaging is weighted by the volume each particle takes up, rather than just the number of particles as in the case of a number-average. This graph presents the average cluster size segmented for all micrographs taken from emulsions emulsified for specific times. The cluster radius depends on whether the sizes were normalized by volume (red) or by number (blue). Both trends show that the cluster size at 25 min is less than that initially, but they take different routes. The blue curve shows the clusters gradually getting smaller with time, whereas the red curve shows cluster growth before they are finally broken down. The reason for this is as follows: at all times there are a number of small clusters, bringing the average down. At 10–20 min a very small number of very large objects can be seen. These sometimes occur at the edge of a jagged boundary between oil and water, and sometimes are elongated and rounded. They look like the remnants of a large droplet folded over many times. These cluster sizes were segmented from low magnification data. The larger area of view allows better statistics. Error bars are the standard error. The conclusion that clusters generally break up with time is broadly in agreement with (Binks et al., 2010) although the behavior for our compositions is markedly more complex.

**Figure 6:**
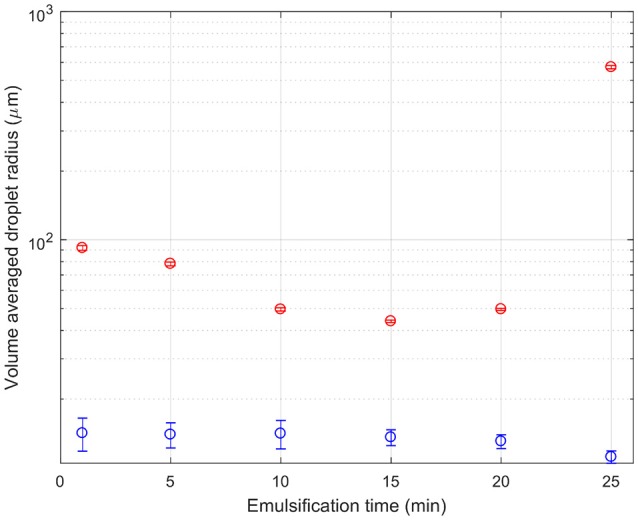
A composite graph of the droplet size as a function of emulsification time. The red symbols indicate the volume average of the size of droplets which are above 20 μm in size as determined from the ×10 confocal micrographs. The blue symbols indicate the volume average of the size of droplets which are below 20 μm in size as determined from the ×40 confocal micrographs.

Figure 6 shows quantitative changes in droplet sizes with emulsification time. The droplet sizes are normalized by volume, and the droplet radius is given. Since there were two data sets with different magnification, the large and small droplets come from the images with the most suitable magnification. The threshold between small (blue) and large (red) droplets is taken as a radius of 20 μm. The two sets of droplets follow different trends. The small droplets begin small, and get smaller and more numerous. Large droplets initially get broken up too. Around 15 min they stop shrinking and instead begin to grow. This is curious and unexpected and is discussed further below.

### Rheology

Several rheology studies have already explored the flow properties of Pickering emulsions fabricated from a range of ingredients (Arditty et al., 2005; Binks et al., 2005; Wolf et al., 2007; Frith et al., 2008; Braisch et al., 2009; Hermes and Clegg, 2013; Katepalli et al., 2017). Early studies focused on systems which had a yield stress at relatively low volume fractions due to the influence of strong attractive interactions between droplets. Unlike with many surfactant-stabilized emulsions, a shear thickening signature was observed at high volume fraction (Wolf et al., 2007). More recently, rheology was combined with imaging for attractive and repulsive Pickering droplets (Hermes and Clegg, 2013) while the flow properties of droplets stabilized by fumed silica have now been explored in some detail (Katepalli et al., 2017) at least in the presence of salt.

**Figure 7:**
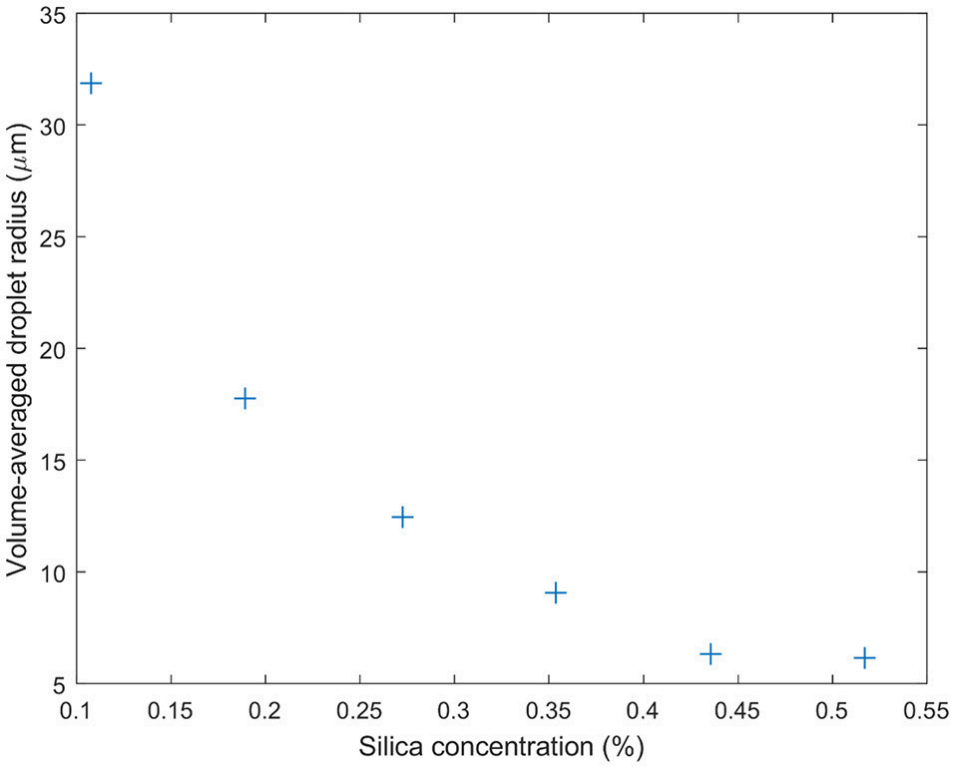
Droplet sizes determined using bright-field microscopy for the emulsions designed for rheology studies. The volume average of the water droplet radius is found to steadily decrease with the concentration of silica particles. The particles were pre-dispersed in the limonene oil phase which occupied 30 vol.% of the sample.

The emulsions prepared for the rheology experiments here needed to be stable with respect to shear. Additionally we wanted to avoid large silica clusters to allow us to study the effect of droplet size alone on the flow of an emulsion (see Methods). Silica was dispersed in limonene with its concentration controlling the droplet size. This stock solution was then diluted in order to make a 70% oil fraction emulsion. Figure 7 illustrates the range of droplet sizes achieved by this route, ranging from around 6–31 μm.

**Figure 8:**
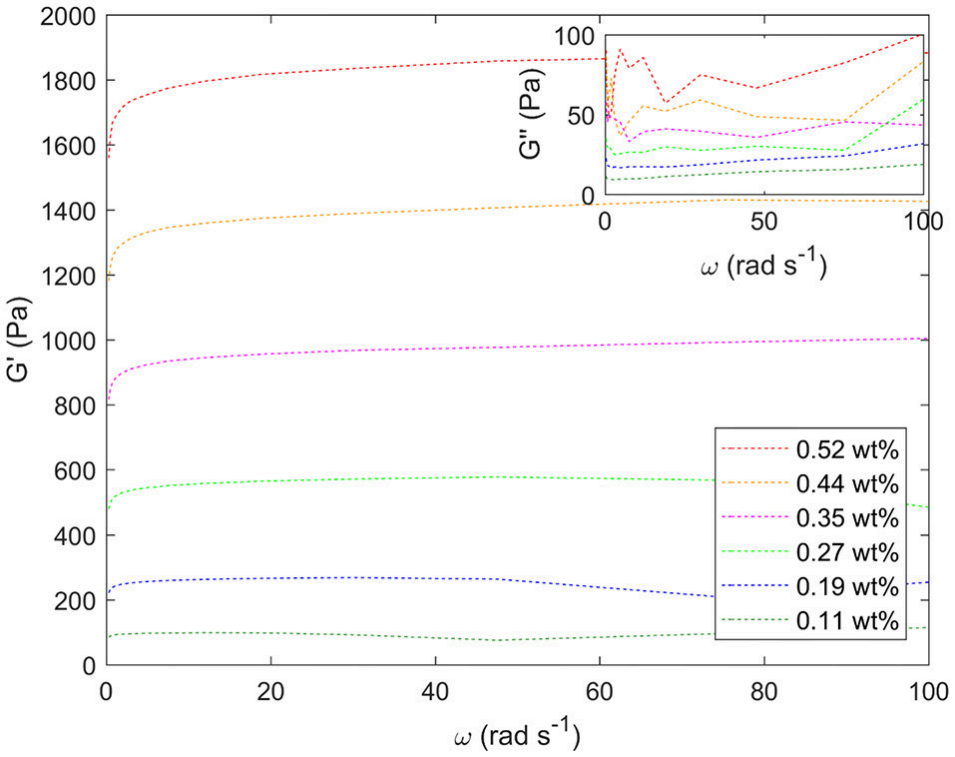
Storage moduli (G') for a series of different water-in-limonene Pickering emulsion samples determined for a frequency (ω) sweep at a strain of 0.6 carried out using a Couette geometry. Inset: the corresponding loss moduli (G”). The storage moduli increase systematically with increasing particle concentration / decreasing droplet size. Weight percentages of silica are given in the legend.

**Figure 9:**
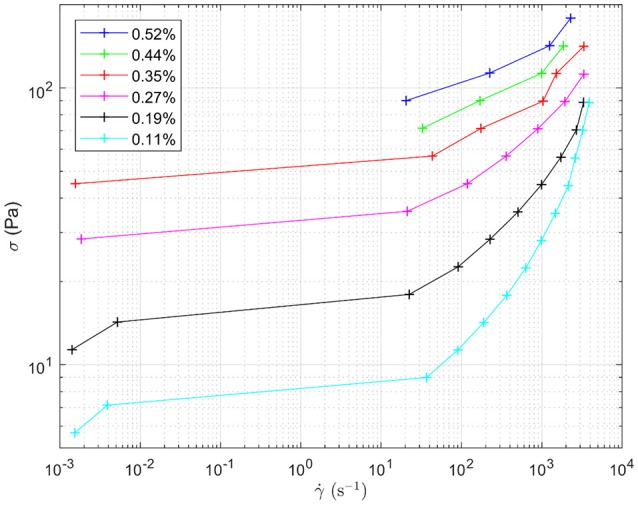
Flow curves for the different water-in-limonene Pickering emulsion samples. The shape of the curve is indicative of the samples having a yield stress which is overcome at higher shear rates. Shear stress (σ) is plotted against shear rate ($\dot{\gamma }$).The legend details the concentration of silica within each emulsion, which controls the size distribution of the droplets making up each emulsion respectively.

Figure 8 shows the response to oscillating shear of a range of emulsions with varying droplet size. In the main graph, the effect of changing droplet size on the storage modulus, G', can be seen for angular velocities between 0 and 100 rad s^−1^. Decreasing the droplet size systematically increases the storage modulus. For example, at intermediate angular frequencies the storage modulus increases from ~100 to 1,800 Pa as the droplet size decreases, Figure 7. The smaller the droplet radius, the more elastic the droplets are. This makes it more difficult for the system to flow. The corresponding loss moduli can be seen inset. The absolute values of the loss moduli are small compared to the storage moduli. With the same range of emulsions, the loss modulus increase is just under an order of magnitude. Figure 9 shows flow curves (the change in shear rate with varying shear stress) for the range of different emulsions with differing droplet sizes. Larger droplets show lower yield stresses. At higher shear rates, the larger droplets have a stronger dependence between the stress and the shear rate in comparison to the more gentle slopes for the smaller droplets. This relationship has been seen before for surfactant-stabilized emulsions (Pal, 1996, 1998).

### Role of emulsification time

Considering together the flow properties (Figures 8, 9) and the evolution of the droplet size (Figure 6), some light is shed upon the underlying mechanism of phase inversion for our composition range. At short times, droplets of all sizes are being broken up as the macroscopic domains and rough emulsion droplets become the droplet size distribution observed in our images. Curiously, at around 15 min of emulsification the larger droplets stop shrinking while the small droplets are becoming less flowable. Indeed the regions of dense droplets in the sample are likely to have a significant yield stress. The break-up and deformation of the larger droplets is the easiest way for the system to flow, however, break-up into smaller droplets appears not to be happening with any permanence. Instead we see these large droplets growing and becoming increasingly folded and deformed. The folding is evident in the growth of new, less bright, silica clusters leading to the apparent increased cluster size in the volume averaged curve in Figure 5. Ultimately, the large population of droplets appear to develop progressively into the continuous phase of a multiple emulsion.

We suggest that the large population of water droplets develops a separate mode of behavior due to exhaustion of the supply of particles. It appears that most particles are: either bound up on the interfaces of a robust population of small droplets or formed into new particle clusters when large droplets are distorted and fold. At the same time, the dense population of small droplets and everything bound around them does not easily flow. By contrast the large droplets can be broken up and deformed easily. Following break up they also rapidly coalesce, giving rise to the many examples of arrested coalescence which are evident in Figures 2, 3. (It is possible that in some cases the droplets form particle bridges, in place of coalescence, although this is difficult to diagnose for the particle sizes we are using here).

This process of multiple emulsion formation is quite different from that observed by Binks and coworkers (Binks et al., 2010). Those authors worked with a range of particle surface chemistries but their studies included a focus on the water/limonene system we have used here. They show that, for an equal volume of water and limonene, the system forms an oil-continuous emulsion at short times but that the water droplets become larger, on average, as mixing continues. They suggest that the sharp increase in droplet size is due to the inclusion of oil droplets within the water droplets, i.e., the opposite type of multiple emulsion to that which we observe. Finally, these large multiple emulsion droplets invert to give a water-continuous simple emulsion.

It is also important to consider whether the behavior we observe could be due to a change in wettability of particle clusters with mixing time. Binks suggested that the changes in emulsion state with increasing duration of high-shear mixing could be due to the improved dispersal of particle clusters (Binks et al., 2010). We have demonstrated here that these clusters exist and that they are broken down during mixing to some extent. While we cannot rule out that two populations of particles exist or that some clusters include a trapped solvent (Clegg et al., 2016) it is far from obvious that the interfaces of the small and large droplets are in anyway different. There is no immediate suggestion that there are two classes of interface leading to a variation in the sign of the mean curvature of the interfaces.

## Conclusions

Using bulk observations, confocal microscopy and rheology we have explored the behavior of Pickering emulsions comprised of water, limonene and fumed silica as a function of mixing time. From a very early stage the samples are completely comprised of water droplets. With continuing mixing time, particle clusters break up and the droplet sizes decrease. As the water droplets break up a subset of large droplets is left behind and some of these even grow. Most of the sample is a dense rigid population of small water droplets and we separately find that such emulsions become increasingly gel-like as the droplet size decreases. By contrast, the remaining larger droplets are floppy and unstable. During the continuing mixing, the large droplets are never permanently broken down to the typical small droplet size. We argue that this is because the supply of accessible particles has been exhausted. Instead large floppy water droplets with low Laplace pressures are folded over on themselves and become crumpled leading to some new (but less dense) particle clusters being formed. Eventually the large water droplets coalesce to form a continuous phase around limonene droplets which contain a dense population of small water droplets.

## Author contributions

Mixing time and confocal microscopy studies were carried out by LS under the guidance of PC. Rheology studies were carried out by LS and KB under the guidance of DH. The manuscript was drafted by PC with contributions from all of the other authors.

### Conflict of interest statement

The authors declare that the research was conducted in the absence of any commercial or financial relationships that could be construed as a potential conflict of interest.
